# Supplementary material for: Mutant huntingtin impairs neurodevelopment in human brain organoids through CHCHD2-mediated neurometabolic failure
Source: Nat Commun. 2024 Aug 22;15:7027. doi: 10.1038/s41467-024-51216-w (PMC11341898; doi:10.1038/s41467-024-51216-w)
Supplement: Supplementary file 1 — Supplementary Information [file 41467_2024_51216_MOESM1_ESM.pdf]

SUPPLEMENTARY INFORMATION

Mutant huntingtin impairs neurodevelopment in human brain organoids through CHCHD2-mediated neurometabolic failure

Lisowski, Lickfett et al.

Manuscript correspondence:

Alessandro Prigione, alessandro.prigione@hhu.de

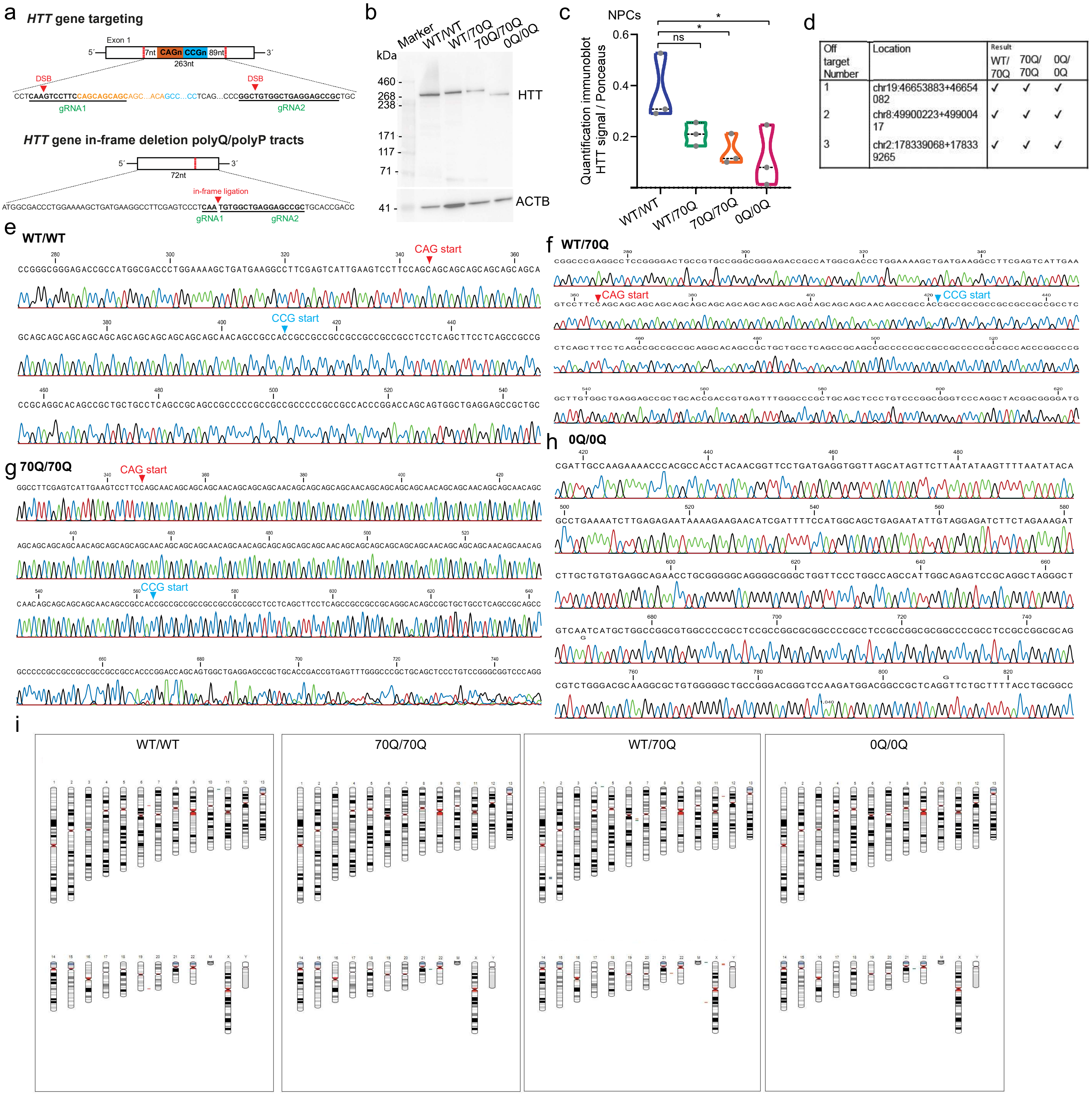
a
HTT gene targeting
Exon 1
5´
7nt
CAGn
CCGn
89nt
3´
263nt
DSB
DSB
gRNA1
gRNA2
HTT gene in-frame deletion polyQ/polyP tracts
72nt
in-frame ligation
b
Marker
WT/WT
WT/70Q
70Q/70Q
0Q/0Q
kDa
460
268
238
171
117
71
41
HTT
ACTB
c
NPCs
ns
*
*
Quantification immunoblot HTT signal / Ponceaus
0.6
0.4
0.2
WT/WT
WT/70Q
70Q/70Q
0Q/0Q
d
Off target Number
Location
Result
WT/ 70Q
70Q/ 70Q
0Q/ 0Q
1
chr19:46653883+46654082
✓
✓
✓
2
chr8:49900223+49900417
✓
✓
✓
3
chr2:178339068+178339265
✓
✓
✓
e
WT/WT
CAG start
CCG start
f
WT/70Q
CAG start
CCG start
g
70Q/70Q
CAG start
CCG start
h
0Q/0Q
i
WT/WT
70Q/70Q
WT/70Q
0Q/0Q

Supplementary Fig. 1. Characterization of engineered isogenic iPSCs carrying mHTT (related to Fig. 1). a Schematics of *HTT* gene targeting. DSB: double-strand breaks, gRNAs: guide RNAs. **b** Representative immunoblot of engineered isogenic NPCs using anti-Huntingtin antibody (ab109115). The results were confirmed in 3 independent experiments. **c** Quantification of HTT immunoblot in NPCs. n=3 independent biological replicates (dots) per line; *p<0.05, ns: not significant; unpaired two-tailed Welch t test. **d** Off-target analysis of top three predicted sites by CRISPOR. Analysis in the engineered iPSC lines (WT/70Q, 70Q/70Q, 0Q/0Q) did not show sequence alterations. **e-h** Sanger sequencing of target *HTT* region including indication of CAG starting site (in red) and CCG starting site (in blue). Images show the wild-type allele for WT/WT and WT/70Q iPSCs, one mutant allele for 70Q/70Q iPSCs, and one allele for 0Q/0Q iPSCs. **i** Normal karyotype assessed by SNP karyotyping (visualized with KaryoStudio v1.4) in WT/WT iPSCs and in the derived engineered lines (WT/70Q, 70Q/70Q, 0Q/0Q).

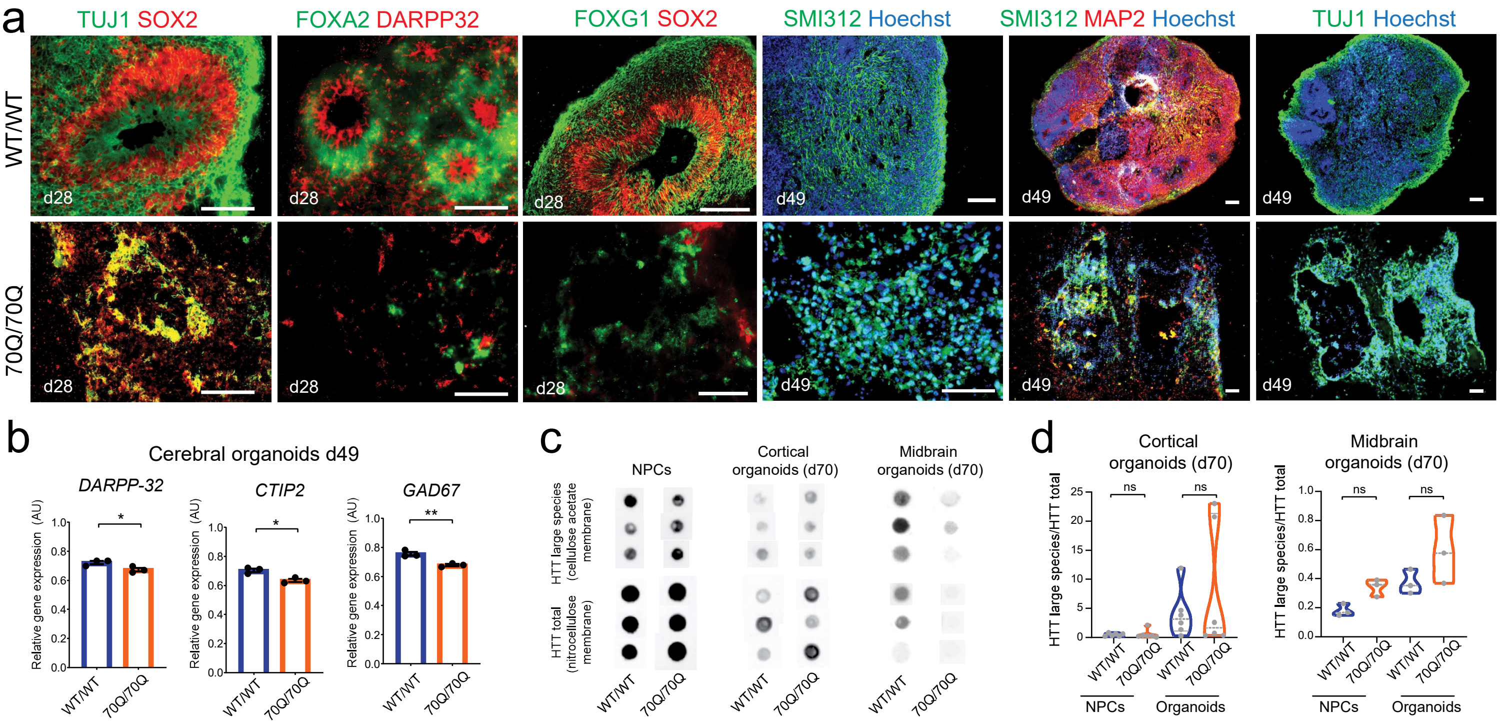

a
TUJ1 SOX2
FOXA2 DARPP32
FOXG1 SOX2
SMI312 Hoechst
SMI312 MAP2 Hoechst
TUJ1 Hoechst
WT/WT
70Q/70Q
d28
d49
b
Cerebral organoids d49
DARPP-32
CTIP2
GAD67
Relative gene expression (AU)
WT/WT
70Q/70Q
c
NPCs
Cortical organoids (d70)
Midbrain organoids (d70)
HTT large species (cellulose acetate membrane)
HTT total (nitrocellulose membrane)
d
Cortical organoids (d70)
Midbrain organoids (d70)
HTT large species/HTT total
ns
NPCs
Organoids

Supplementary Fig. 2. Analysis of brain organoids carrying mHTT (related to Fig. 1 and Fig. 2). a Immunostaining of unguided cerebral organoids at day 28 and 49 showing defective cytoarchitecture and neural progenitor cell (NPC) organization in 70/70Q. Data were repeated in three independent experiments. Scale bar: 100 µm. **b** qPCR analysis of neuronal markers in unguided cerebral organoids at day 49. Mean +/- s.e.m.; n=3 independent biological replicates (dots) per line (AU=arbitrary units); *p<0.05 **p<0.01; unpaired two-tailed t test. Four organoids were pooled for each individual RNA isolation. **c-d** Representative images and related quantification of filter retardation assay (FRA) in NPCs and in guided region-specific brain organoids (cortical and midbrain) to assess the potential presence of HTT protein aggregates using anti-Huntingtin antibody (ab109115). Cellulose acetate membranes were used to visualize aggregated HTT protein species, and nitrocellulose membrane to visualize total HTT protein species. n=3 independent biological replicates (dots) per line; ns: not significant; unpaired two-tailed t test.

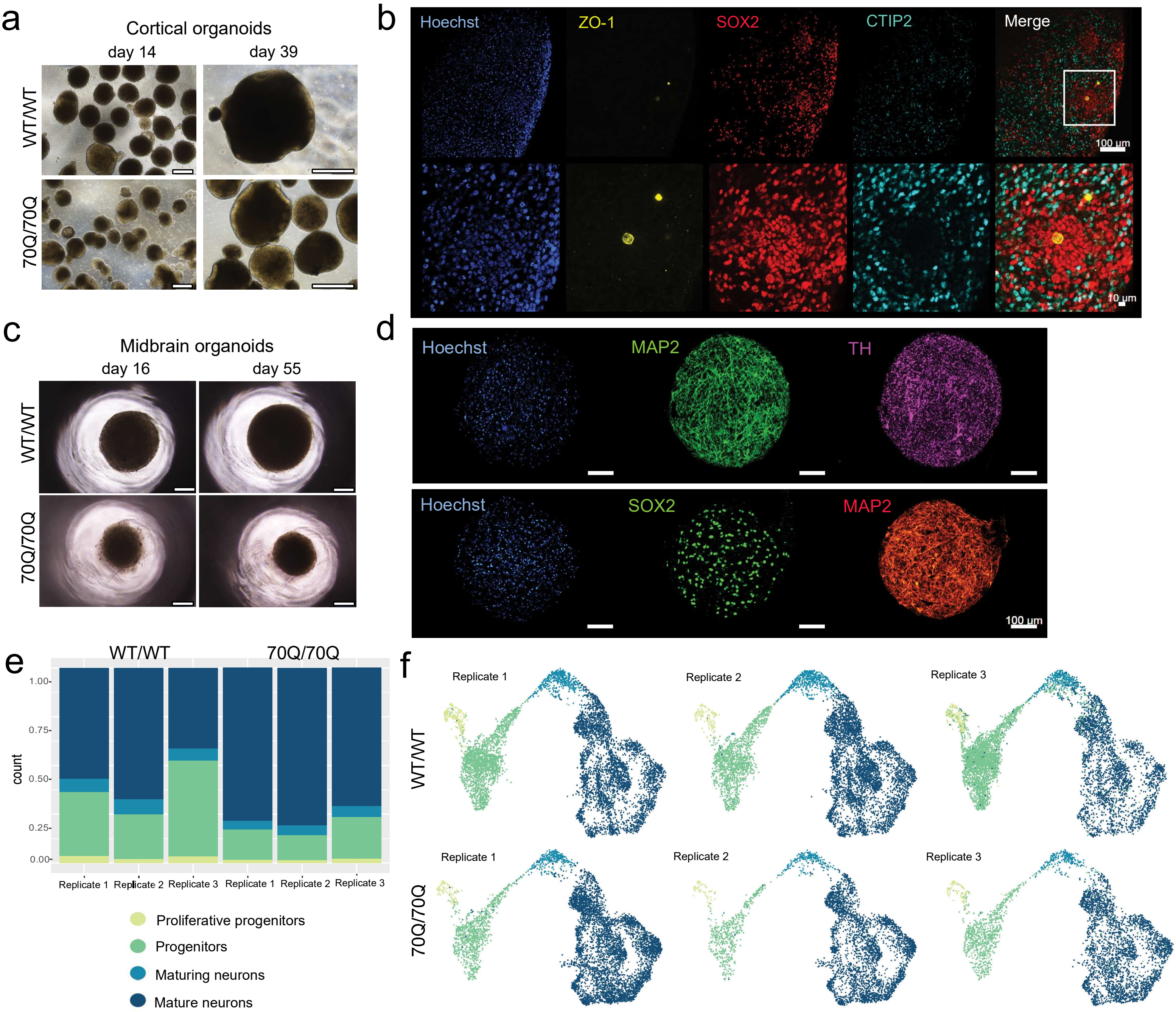

a
Cortical organoids
day 14
day 39
WT/WT
70Q/70Q
b
Hoechst
ZO-1
SOX2
CTIP2
Merge
100 µm
10 µm
c
Midbrain organoids
day 16
day 55
WT/WT
70Q/70Q
d
Hoechst
MAP2
TH
Hoechst
SOX2
MAP2
100 µm
e
WT/WT
70Q/70Q
count
1.00
0.75
0.50
0.25
0.00
Replicate 1
Replicate 2
Replicate 3
Replicate 1
Replicate 2
Replicate 3
Proliferative progenitors
Progenitors
Maturing neurons
Mature neurons
f
Replicate 1
Replicate 2
Replicate 3
WT/WT
Replicate 1
Replicate 2
Replicate 3
70Q/70Q

Supplementary Fig. 3. Guided region-specific brain organoids carrying mHTT (related to Fig. 2). a Representative images of cortical organoids visualized with bright field microscope. Data were repeated in three independent experiments. Scale bar: 400 µm. **b** Immunostaining of cortical organoids at day 35 showing the presence of progenitor and neuronal and cortical markers. Data were repeated in two independent experiments. Scale bar: 100 µm (above), 10 µm (below). **c** Representative images of midbrain organoids visualized with bright field microscope. Data were repeated in three independent experiments. Scale bar: 200 µm. **d** Immunostaining of midbrain organoids at day 35 showing the presence of progenitor and neuronal and dopaminergic markers. Data were repeated in two independent experiments. Scale bar: 100 µm. **e** Single-cell RNA sequencing-based quantification of the four annotated cell populations in three biological replicates (each containing 48 individual organoids) of midbrain organoids from WT/WT and 70Q/70Q. **f** UMAP analysis showing the overall cell composition of the four populations distribution in biological replicates of midbrain organoids from WT/WT and 70Q/70Q.

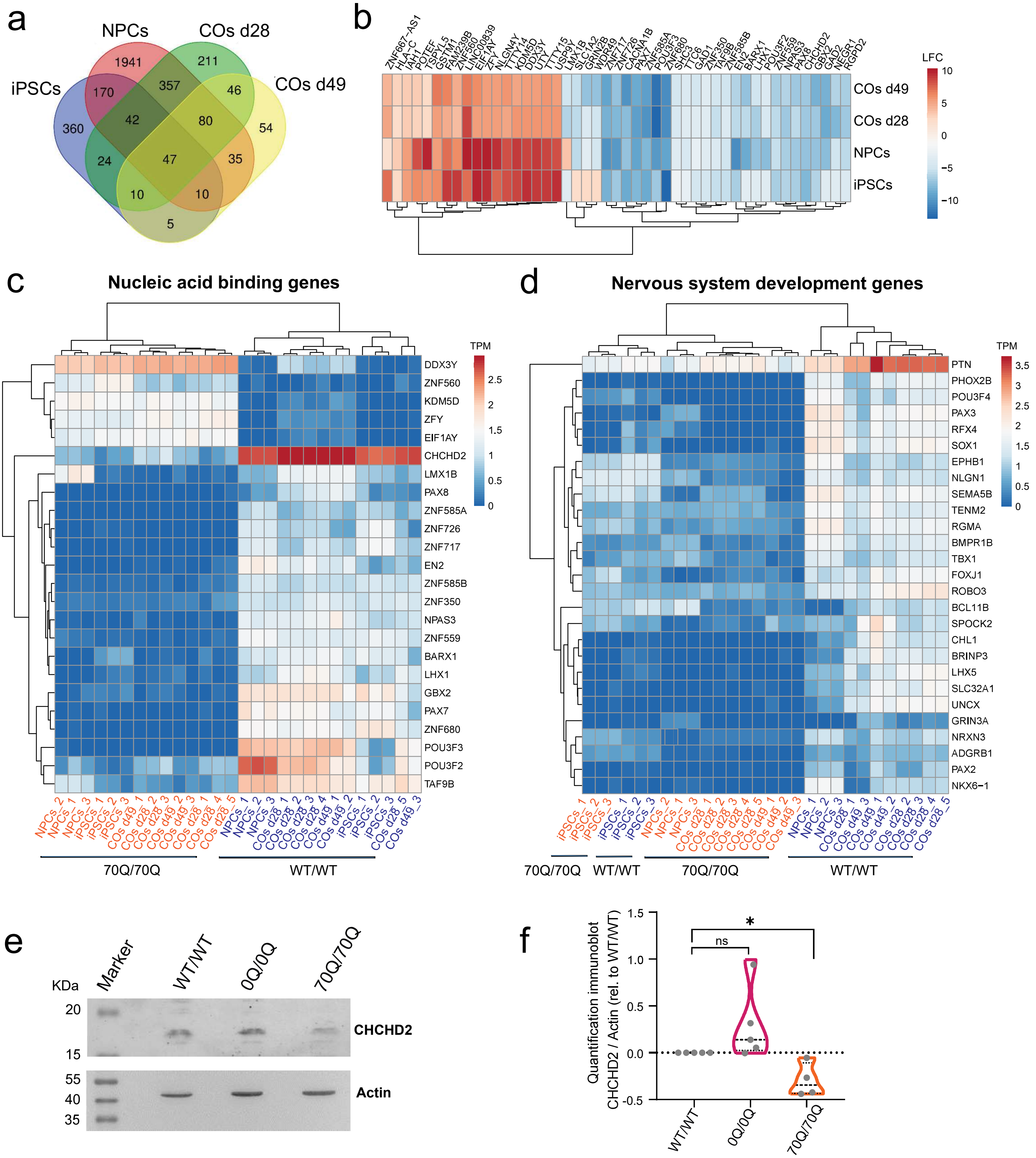
a
NPCs
COs d28
iPSCs
COs d49
1941
211
170
357
46
360
42
80
54
24
47
35
10
10
5
b
LFC
COs d49
COs d28
NPCs
iPSCs
c
Nucleic acid binding genes
TPM
DDX3Y
ZNF560
KDM5D
ZFY
EIF1AY
CHCHD2
LMX1B
PAX8
ZNF585A
ZNF726
ZNF717
EN2
ZNF585B
ZNF350
NPAS3
ZNF559
BARX1
LHX1
GBX2
PAX7
ZNF680
POU3F3
POU3F2
TAF9B
70Q/70Q
WT/WT
d
Nervous system development genes
TPM
PTN
PHOX2B
POU3F4
PAX3
RFX4
SOX1
EPHB1
NLGN1
SEMA5B
TENM2
RGMA
BMPR1B
TBX1
FOXJ1
ROBO3
BCL11B
SPOCK2
CHL1
BRINP3
LHX5
SLC32A1
UNCX
GRIN3A
NRXN3
ADGRB1
PAX2
NKX6-1
70Q/70Q
WT/WT
70Q/70Q
WT/WT
e
Marker
WT/WT
0Q/0Q
70Q/70Q
KDa
20
15
55
40
35
CHCHD2
Actin
f
Quantification immunoblot
CHCHD2 / Actin (rel. to WT/WT)
ns
*
WT/WT
0Q/0Q
70Q/70Q

Supplementary Fig. 4. Transcriptional signatures of engineered mHTT cells across different neurodevelopmental stages (related to Fig. 3). a Venn diagram of unique and common transcripts among iPSCs, NPCs, cerebral organoids (COs) at day 28, and COs at day 49. **b** Heatmap of log-fold change (LFC) comparisons of differential gene expression of the 47 transcripts in common across all neurodevelopmental stages in 70/70Q cells compared to WT/WT cells. **c-d** Heatmap of the normalized expression values (TPM: Transcripts Per Million) of transcripts belonging to the GO category of “nucleic acid binding” and “nervous system development”. **e-f** Representative immunoblot and related quantification of CHCHD2 expression and beta actin in NPCs. n=3 independent biological replicates (dots) per line; $^*p<0.05$; ns: not significant; unpaired two-tailed Welch t test.

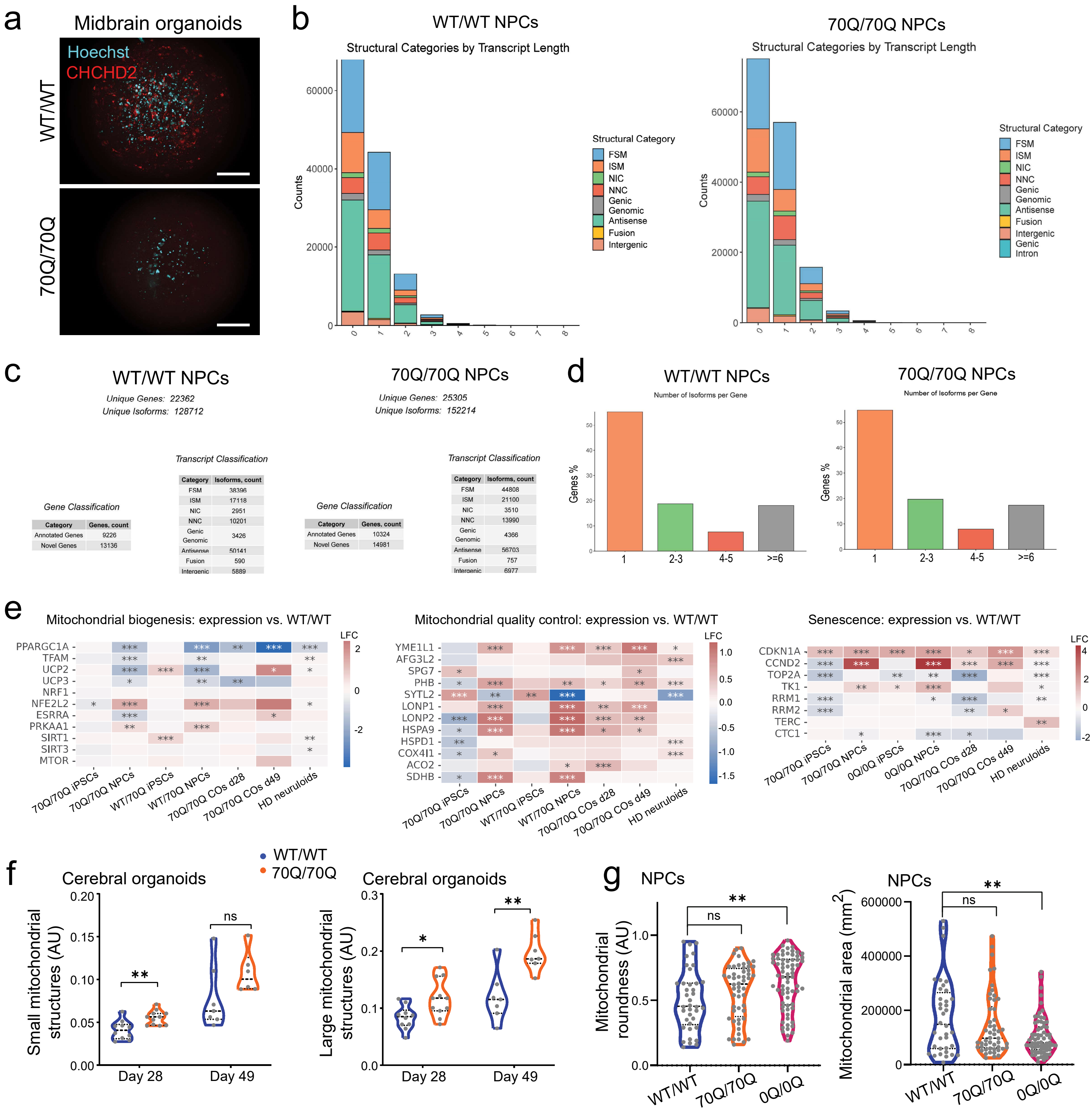

a
Midbrain organoids
Hoechst
CHCHD2
WT/WT
70Q/70Q
b
WT/WT NPCs
Structural Categories by Transcript Length
70Q/70Q NPCs
Structural Categories by Transcript Length
Counts
Structural Category
FSM
ISM
NIC
NNC
Genic Genomic
Antisense
Fusion
Intergenic
Genic Intron
c
WT/WT NPCs
Unique Genes: 22362
Unique Isoforms: 128712
Gene Classification
Category | Genes, count
Annotated Genes | 9226
Novel Genes | 13136
Transcript Classification
Category | Isoforms, count
FSM | 38396
ISM | 17118
NIC | 2951
NNC | 10201
Genic Genomic | 3426
Antisense | 50141
Fusion | 590
Intergenic | 5889
70Q/70Q NPCs
Unique Genes: 25305
Unique Isoforms: 152214
Gene Classification
Category | Genes, count
Annotated Genes | 10324
Novel Genes | 14981
Transcript Classification
Category | Isoforms, count
FSM | 44808
ISM | 21100
NIC | 3510
NNC | 13990
Genic Genomic | 4366
Antisense | 56703
Fusion | 757
Intergenic | 6977
d
WT/WT NPCs
Number of Isoforms per Gene
70Q/70Q NPCs
Number of Isoforms per Gene
Genes %
1
2-3
4-5
>=6
e
Mitochondrial biogenesis: expression vs. WT/WT
PPARGC1A
TFAM
UCP2
UCP3
NRF1
NFE2L2
ESRRA
PRKAA1
SIRT1
SIRT3
MTOR
LFC
Mitochondrial quality control: expression vs. WT/WT
YME1L1
AFG3L2
SPG7
PHB
SYTL2
LONP1
LONP2
HSPA9
HSPD1
COX4I1
ACO2
SDHB
Senescence: expression vs. WT/WT
CDKN1A
CCND2
TOP2A
TK1
RRM1
RRM2
TERC
CTC1
70Q/70Q iPSCs
70Q/70Q NPCs
WT/70Q iPSCs
WT/70Q NPCs
0Q/0Q iPSCs
0Q/0Q NPCs
70Q/70Q COs d28
70Q/70Q COs d49
HD neuruloids
f
Cerebral organoids
WT/WT
70Q/70Q
Small mitochondrial structures (AU)
Large mitochondrial structures (AU)
Day 28
Day 49
ns
g
NPCs
Mitochondrial roundness (AU)
Mitochondrial area (mm2)
WT/WT
70Q/70Q
0Q/0Q

Supplementary Fig. 5. Mitochondria morpho-dynamics changes in neural cells carrying mHTT (related to Fig. 4 and Fig. 5). a Representative images of midbrain organoids from WT/WT and 70/70Q stained with CHCHD2. Data were repeated in two independent experiments. Scale bar: 100 µm. **b-d** Long-read sequencing analysis showing the distribution of the recognized variants classified by structural categories, the number of isoforms detected, and the distribution of number of isoforms per gene in WT/WT NPCs and 70Q/70Q NPCs. **e** Heatmap of log-fold change (LFC) comparisons of genes involved in mitochondrial biogenesis, mitochondrial quality control, and senescence based on bulk RNA sequencing from iPSCs, NPCs, and cerebral organoids (COs) derived from 70Q/70Q. HD neuruloids were compared to WT neuruloids. **f** Quantification of immunostaining for mitochondrial footprint of small mitochondrial structures (<0.5 µm) and large mitochondrial structures (>0.5 µm) based on TOM20 signal in cerebral organoids from 70Q/70Q and WT/WT. Dots represent individual images collected over three independent experiments (AU=arbitrary units). *p<0.05, **p<0.01, ***p<0.001, ns: not significant; unpaired two-tailed Welch t test. **g** Quantification of mitochondrial roundness and mitochondrial area based on electron microscope images. Each dot represents an individual mitochondrion from NPCs measured in two biological replicates per line (AU=arbitrary units). **p<0.01, ns: not significant; unpaired two-tailed Welch t test.

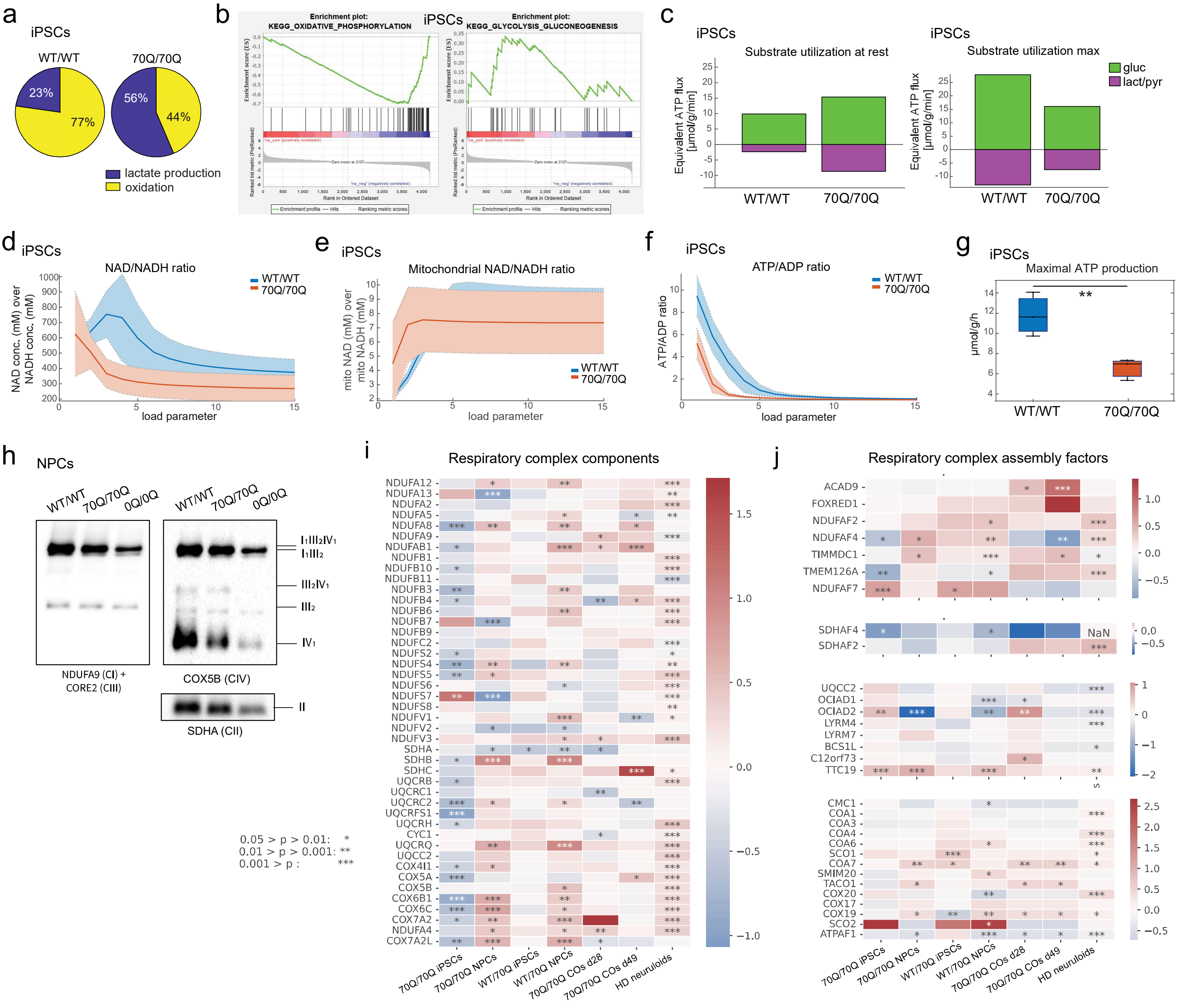
a
iPSCs
WT/WT
70Q/70Q
23%
77%
56%
44%
lactate production
oxidation
b
iPSCs
Enrichment plot: KEGG_OXIDATIVE_PHOSPHORYLATION
Enrichment plot: KEGG_GLYCOLYSIS_GLUCONEOGENESIS
Enrichment score (ES)
Ranked list metric (PreRanked)
Rank in Ordered Dataset
Enrichment profile — Hits — Ranking metric scores
c
iPSCs
Substrate utilization at rest
Substrate utilization max
Equivalent ATP flux [µmol/g/min]
gluc
lact/pyr
WT/WT
70Q/70Q
d
iPSCs
NAD/NADH ratio
NAD conc. (mM) over NADH conc. (mM)
load parameter
WT/WT
70Q/70Q
e
iPSCs
Mitochondrial NAD/NADH ratio
mito NAD (mM) over mito NADH (mM)
load parameter
WT/WT
70Q/70Q
f
iPSCs
ATP/ADP ratio
ATP/ADP ratio
load parameter
WT/WT
70Q/70Q
g
iPSCs
Maximal ATP production
µmol/g/h
**
WT/WT
70Q/70Q
h
NPCs
WT/WT
70Q/70Q
0Q/0Q
NDUFA9 (CI) + CORE2 (CIII)
COX5B (CIV)
SDHA (CII)
I1III2IV1
I1III2
III2IV1
III2
IV1
II
0.05 > p > 0.01: *
0.01 > p > 0.001: **
0.001 > p : ***
i
Respiratory complex components
NDUFA12
NDUFA13
NDUFA2
NDUFA5
NDUFA8
NDUFA9
NDUFAB1
NDUFB1
NDUFB10
NDUFB11
NDUFB3
NDUFB4
NDUFB6
NDUFB7
NDUFB9
NDUFC2
NDUFS2
NDUFS4
NDUFS5
NDUFS6
NDUFS7
NDUFS8
NDUFV1
NDUFV2
NDUFV3
SDHA
SDHB
SDHC
UQCRB
UQCRC1
UQCRC2
UQCRFS1
UQCRH
CYC1
UQCRQ
UQCC2
COX4I1
COX5A
COX5B
COX6B1
COX6C
COX7A2
NDUFA4
COX7A2L
70Q/70Q iPSCs
70Q/70Q NPCs
WT/70Q iPSCs
WT/70Q NPCs
70Q/70Q COs d28
70Q/70Q COs d49
HD neuruloids
j
Respiratory complex assembly factors
ACAD9
FOXRED1
NDUFAF2
NDUFAF4
TIMMDC1
TMEM126A
NDUFAF7
SDHAF4
SDHAF2
NaN
UQCC2
OCIAD1
OCIAD2
LYRM4
LYRM7
BCS1L
C12orf73
TTC19
CMC1
COA1
COA3
COA4
COA6
SCO1
COA7
SMIM20
TACO1
COX20
COX17
COX19
SCO2
ATPAF1
70Q/70Q iPSCs
70Q/70Q NPCs
WT/70Q iPSCs
WT/70Q NPCs
70Q/70Q COs d28
70Q/70Q COs d49
HD neuruloids

Supplementary Fig. 6. Disruption of metabolism and mitochondrial respiratory chain complexes in iPSCs and NPCs carrying mHTT (related to Fig. 5). a Relative glucose utilization at resting energy demands in iPSCs from WT/WT and 70Q/70Q based on proteomics-driven functional metabolic analysis. **b** Gene set enrichment analysis (GSEA) showing decreased oxidative phosphorylation (OXPHOS) and increased glycolysis/gluconeogenesis in 70Q/70Q iPSCs compared to WT/WT iPSCs. **c** Proteomics-driven functional metabolic analysis depicting the substrate utilization at maximal energy demands of iPSCs from WT/WT and 70Q/70Q. Energetic capacities were evaluated by computing the changes of metabolic state elicited by an increase of the ATP consumption rate above the resting value. Bar graphs show consumption of glucose (gluc) and production of lactate-pyruvate (lact/pyr). **d-e** Metabolic state variables for NAD/NADH ratio and mitochondrial NAD/NADH ratio in iPSCs from WT/WT and 70Q/70Q in dependence of increasing energetic demands. **f-g** Computed ATP/ADP ratio in dependence of increasing energetic demands and maximal ATP production capacity in iPSCs from WT/WT and 70Q/70Q. **h** Representative blue native PAGE of mitochondrial complex assembly in WT/WT NPCs, 70Q/70Q NPCs, and 0Q/0Q NPCs. n=3 independent biological replicates per line run in two different blots. **i-j** Heatmap of log fold change (LFC) comparisons of genes involved in mitochondrial respiratory chain composition and assembly based on bulk RNA sequencing performed in iPSCs, NPCs, and COs from 70Q/70Q and WT/70Q compared to WT/WT. HD neuruloids were compared to WT neuruloids.

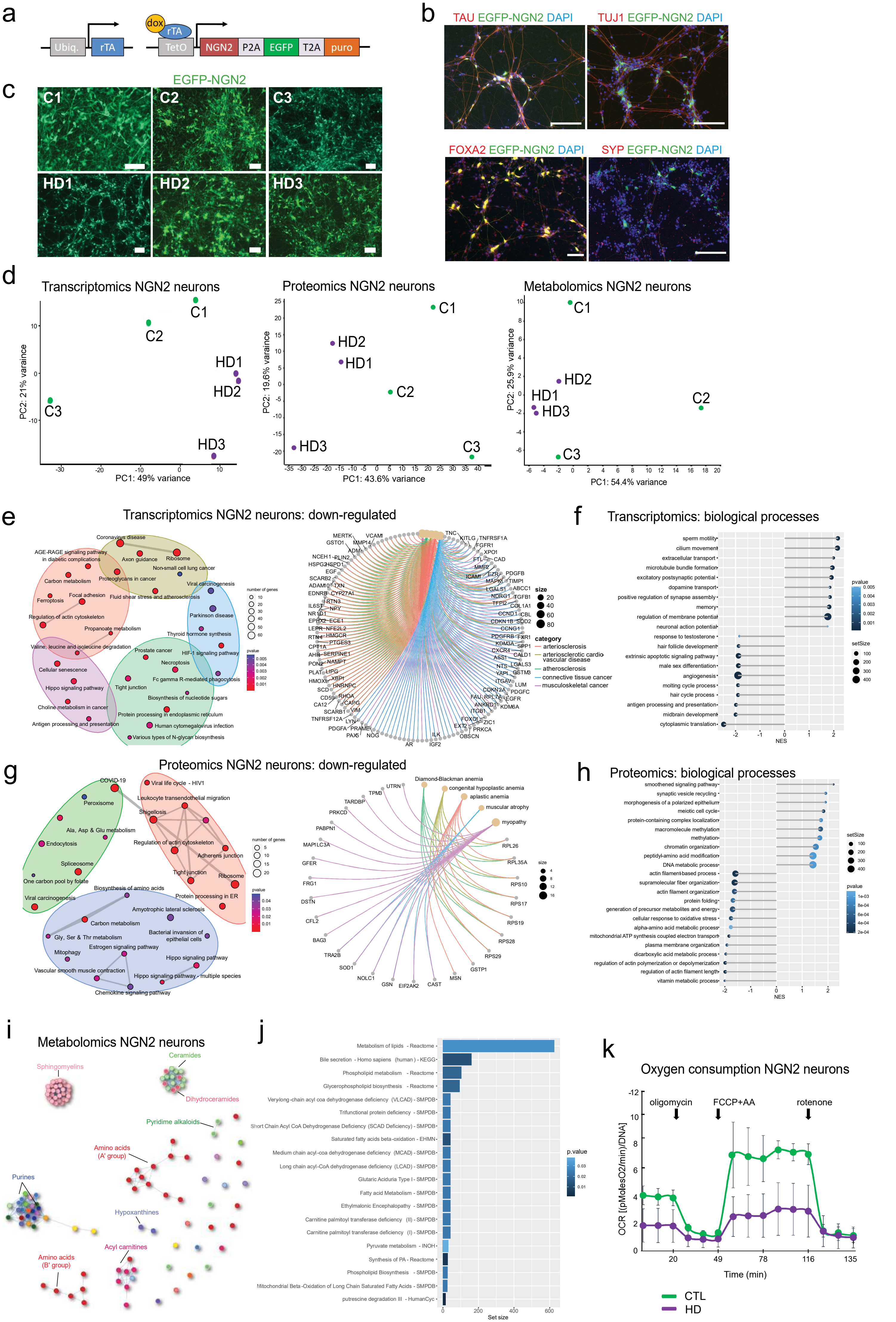

a
Ubiq.
rTA
dox
rTA
TetO
NGN2
P2A
EGFP
T2A
puro
b
TAU EGFP-NGN2 DAPI
TUJ1 EGFP-NGN2 DAPI
FOXA2 EGFP-NGN2 DAPI
SYP EGFP-NGN2 DAPI
c
EGFP-NGN2
C1
C2
C3
HD1
HD2
HD3
d
Transcriptomics NGN2 neurons
PC2: 21% varaince
PC1: 49% variance
Proteomics NGN2 neurons
PC2: 19.6% varaince
PC1: 43.6% variance
Metabolomics NGN2 neurons
PC2: 25.9% variance
PC1: 54.4% variance
e
Transcriptomics NGN2 neurons: down-regulated
Coronavirus disease
AGE-RAGE signaling pathway in diabetic complications
Axon guidance
Ribosome
Non-small cell lung cancer
Carbon metabolism
Proteoglycans in cancer
Viral carcinogenesis
Ferroptosis
Focal adhesion
Fluid shear stress and atherosclerosis
Regulation of actin cytoskeleton
Parkinson disease
Propanoate metabolism
Thyroid hormone synthesis
Valine, leucine and isoleucine degradation
Prostate cancer
HIF-1 signaling pathway
Cellular senescence
Necroptosis
Fc gamma R-mediated phagocytosis
Hippo signaling pathway
Tight junction
Biosynthesis of nucleotide sugars
Choline metabolism in cancer
Protein processing in endoplasmic reticulum
Antigen processing and presentation
Human cytomegalovirus infection
Various types of N-glycan biosynthesis
number of genes
pvalue
size
category
arteriosclerosis
arteriosclerotic cardio vascular disease
atherosclerosis
connective tissue cancer
musculoskeletal cancer
f
Transcriptomics: biological processes
sperm motility
cilium movement
extracellular transport
microtubule bundle formation
excitatory postsynaptic potential
dopamine transport
positive regulation of synapse assembly
memory
regulation of membrane potential
neuronal action potential
response to testosterone
hair follicle development
extrinsic apoptotic signaling pathway
male sex differentiation
angiogenesis
molting cycle process
hair cycle process
antigen processing and presentation
midbrain development
cytoplasmic translation
NES
pvalue
setSize
g
Proteomics NGN2 neurons: down-regulated
COVID-19
Viral life cycle - HIV1
Peroxisome
Leukocyte transendothelial migration
Shigellosis
Ala, Asp & Glu metabolism
Endocytosis
Regulation of actin cytoskeleton
Adherens junction
Spliceosome
Tight junction
Ribosome
One carbon pool by folate
Biosynthesis of amino acids
Viral carcinogenesis
Protein processing in ER
Amyotrophic lateral sclerosis
Carbon metabolism
Bacterial invasion of epithelial cells
Gly, Ser & Thr metabolism
Estrogen signaling pathway
Mitophagy
Hippo signaling pathway
Vascular smooth muscle contraction
Hippo signaling pathway - multiple species
Chemokine signaling pathway
Diamond-Blackman anemia
congenital hypoplastic anemia
aplastic anemia
muscular atrophy
myopathy
h
Proteomics: biological processes
smoothened signaling pathway
synaptic vesicle recycling
morphogenesis of a polarized epithelium
meiotic cell cycle
protein-containing complex localization
macromolecule methylation
methylation
chromatin organization
peptidyl-amino acid modification
DNA metabolic process
actin filament-based process
supramolecular fiber organization
actin filament organization
protein folding
generation of precursor metabolites and energy
cellular response to oxidative stress
alpha-amino acid metabolic process
mitochondrial ATP synthesis coupled electron transport
plasma membrane organization
dicarboxylic acid metabolic process
regulation of actin polymerization or depolymerization
regulation of actin filament length
vitamin metabolic process
NES
i
Metabolomics NGN2 neurons
Sphingomyelins
Ceramides
Dihydroceramides
Pyridime alkaloids
Amino acids (A' group)
Purines
Hypoxanthines
Amino acids (B' group)
Acyl carnitines
j
Metabolism of lipids - Reactome
Bile secretion - Homo sapiens (human) - KEGG
Phospholipid metabolism - Reactome
Glycerophospholipid biosynthesis - Reactome
Very-long-chain acyl coa dehydrogenase deficiency (VLCAD) - SMPDB
Trifunctional protein deficiency - SMPDB
Short Chain Acyl CoA Dehydrogenase Deficiency (SCAD Deficiency) - SMPDB
Saturated fatty acids beta-oxidation - EHMN
Medium chain acyl-coa dehydrogenase deficiency (MCAD) - SMPDB
Long chain acyl-CoA dehydrogenase deficiency (LCAD) - SMPDB
Glutaric Aciduria Type I - SMPDB
Fatty acid Metabolism - SMPDB
Ethylmalonic Encephalopathy - SMPDB
Carnitine palmitoyl transferase deficiency (II) - SMPDB
Carnitine palmitoyl transferase deficiency (I) - SMPDB
Pyruvate metabolism - INOH
Synthesis of PA - Reactome
Phospholipid Biosynthesis - SMPDB
Mitochondrial Beta-Oxidation of Long Chain Saturated Fatty Acids - SMPDB
putrescine degradation III - HumanCyc
p.value
Set size
k
Oxygen consumption NGN2 neurons
oligomycin
FCCP+AA
rotenone
OCR [(pMolesO2/min)/DNA]
Time (min)
CTL
HD

Supplementary Fig. 7. Characterization and multi-omics analysis of NGN2 neurons from three healthy controls and three individuals with HD (related to Fig. 6). a Lentiviral constructs used for transducing NPCs for NGN2 neuron derivation. **b** Expression of neuronal and synaptic markers in NGN2 neurons at day 14 of differentiation from NPCs (C1 line). Data were repeated in three independent experiments. Scale bar: 100 µm. **c** NGN2 neurons at day 14 of differentiation from healthy controls (C1, C2, C3) and individuals with HD (HD1, HD2, HD3). Data were repeated in three independent experiments. **d** Principal component analysis (PCA) plots showing the distribution of the NGN2 neurons from healthy controls (C1, C2, C3) and individuals with HD (HD1, HD2, HD3) based on transcriptomics, proteomics, and metabolomics. **e** Transcriptomics analysis of NGN2 neurons. Left: over-representation analysis (ORA) of downregulated DEGs for KEGG pathways; right: ORA of downregulated DEGs for human diseases. **f** GSEA for affected GO biological processes (BPs) (NES: normalized enrichment score). **g** Proteomics analysis of NGN2 neurons. Left: ORA of downregulated DEPs for KEGG pathways; right: ORA of downregulated DEPs for human diseases. **h** GSEA for affected GO BPs (NES: normalized enrichment score). **i** Metabolomics analysis of NGN2 neurons. Structure similarity analysis grouping of the dysregulated metabolites based on their family hierarchy. **j** ORA of DEMs for affected metabolic pathways in multiple databases. **k** Oxygen consumption rate (OCR) profile obtained with Seahorse Bioanalyzer in NGN2 neurons from healthy controls (C1, C2, C3) and individuals with HD (HD1, HD2, HD3). Mean +/- s.e.m.; n=3 independent biological replicates per line.

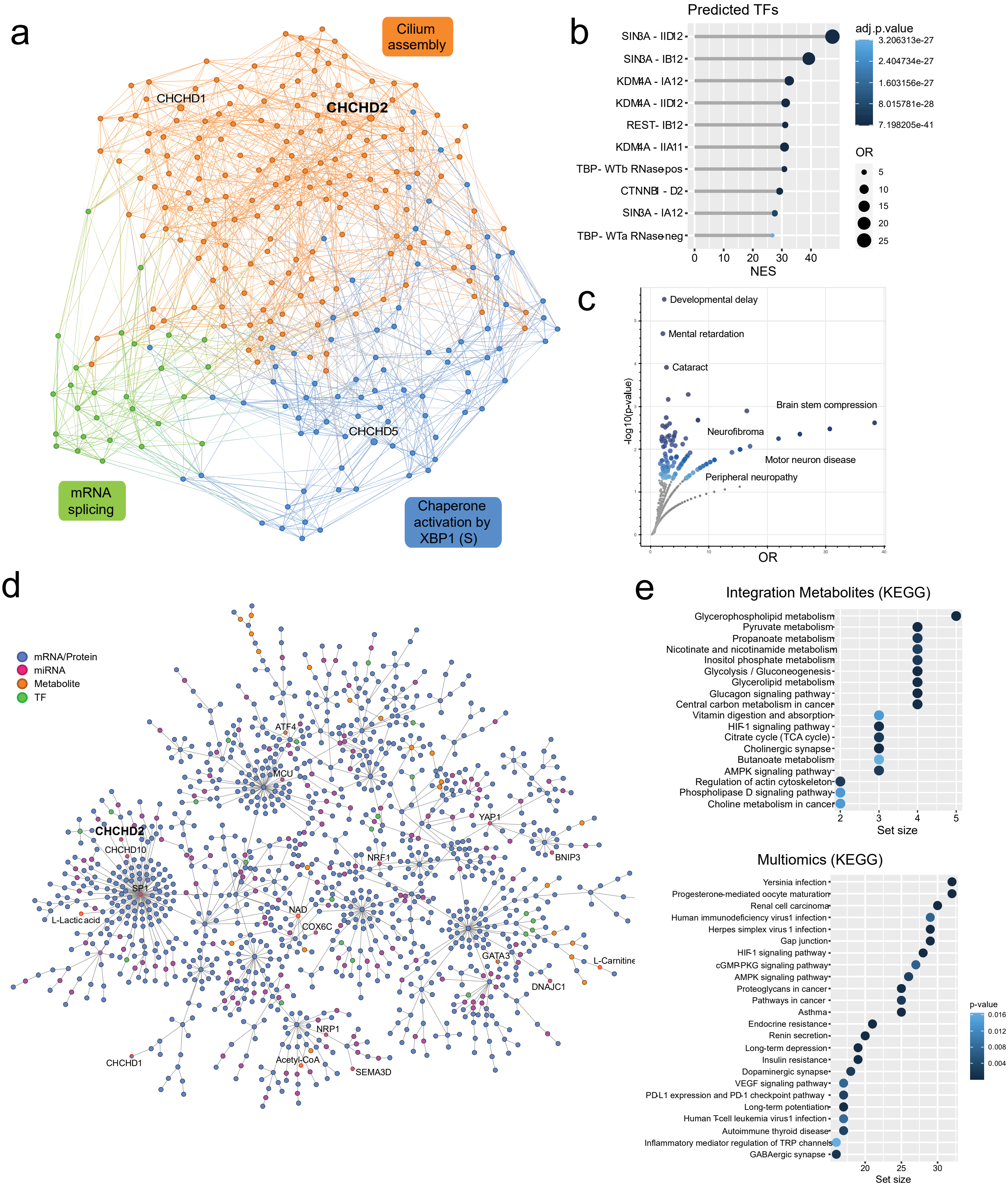
a
Cilium assembly
CHCHD1
CHCHD2
CHCHD5
mRNA splicing
Chaperone activation by XBP1 (S)
b
Predicted TFs
SIN3A - IID12
SIN3A - IB12
KDM4A - IA12
KDM4A - IID12
REST - IB12
KDM4A - IIA11
TBP - WTb RNase pos
CTNNB1 - D2
SIN3A - IA12
TBP - WTa RNase neg
0
10
20
30
40
NES
adj.p.value
3.206313e-27
2.404734e-27
1.603156e-27
8.015781e-28
7.198205e-41
OR
5
10
15
20
25
c
Developmental delay
Mental retardation
Cataract
Brain stem compression
Neurofibroma
Motor neuron disease
Peripheral neuropathy
-log10(p-value)
OR
d
mRNA/Protein
miRNA
Metabolite
TF
ATF4
MCU
CHCHD2
CHCHD10
YAP1
BNIP3
NRF1
SP1
L-Lactic acid
NAD
COX6C
GATA3
L-Carnitine
DNAJC1
NRP1
CHCHD1
Acetyl-CoA
SEMA3D
e
Integration Metabolites (KEGG)
Glycerophospholipid metabolism
Pyruvate metabolism
Propanoate metabolism
Nicotinate and nicotinamide metabolism
Inositol phosphate metabolism
Glycolysis / Gluconeogenesis
Glycerolipid metabolism
Glucagon signaling pathway
Central carbon metabolism in cancer
Vitamin digestion and absorption
HIF-1 signaling pathway
Citrate cycle (TCA cycle)
Cholinergic synapse
Butanoate metabolism
AMPK signaling pathway
Regulation of actin cytoskeleton
Phospholipase D signaling pathway
Choline metabolism in cancer
2
3
4
5
Set size
Multiomics (KEGG)
Yersinia infection
Progesterone-mediated oocyte maturation
Renal cell carcinoma
Human immunodeficiency virus 1 infection
Herpes simplex virus 1 infection
Gap junction
HIF-1 signaling pathway
cGMP-PKG signaling pathway
AMPK signaling pathway
Proteoglycans in cancer
Pathways in cancer
Asthma
Endocrine resistance
Renin secretion
Long-term depression
Insulin resistance
Dopaminergic synapse
VEGF signaling pathway
PD-L1 expression and PD-1 checkpoint pathway
Long-term potentiation
Human T-cell leukemia virus 1 infection
Autoimmune thyroid disease
Inflammatory mediator regulation of TRP channels
GABAergic synapse
20
25
30
Set size
p-value
0.016
0.012
0.008
0.004

Supplementary Fig. 8. Multi-omics integration of NGN2 neurons (related to Fig. 7). a Protein co-expression network in NGN2 neurons showing the biggest cluster of co-expressed proteins (n=272) including CHCH domain-containing proteins (CHCHD1, CHCHD2, CHCHD5). Three modules were generated by breaking the network into communities of densely connected proteins. Enrichment analysis for REACTOME pathways indicated that they are associated with cilium assembly, chaperone activation by XBP1(S) and mRNA splicing. **b** Enrichment analysis for upstream transcription factors of the 272 co-expressed proteins (NES: normalized enrichment score). **c** ORA of 272 co-expressed proteins for human diseases in the GeDiPNet database (OR: odds ratio). **d** Multi-omics integration of NGN2 neurons. Network of significantly dysregulated transcripts, proteins and metabolites along with predicted intermediate miRNAs and transcription factors. **e** Top: enrichment analysis of metabolites included in the multi-omics network. Bottom: enrichment analysis of all entities that participate in the multi-omics network.

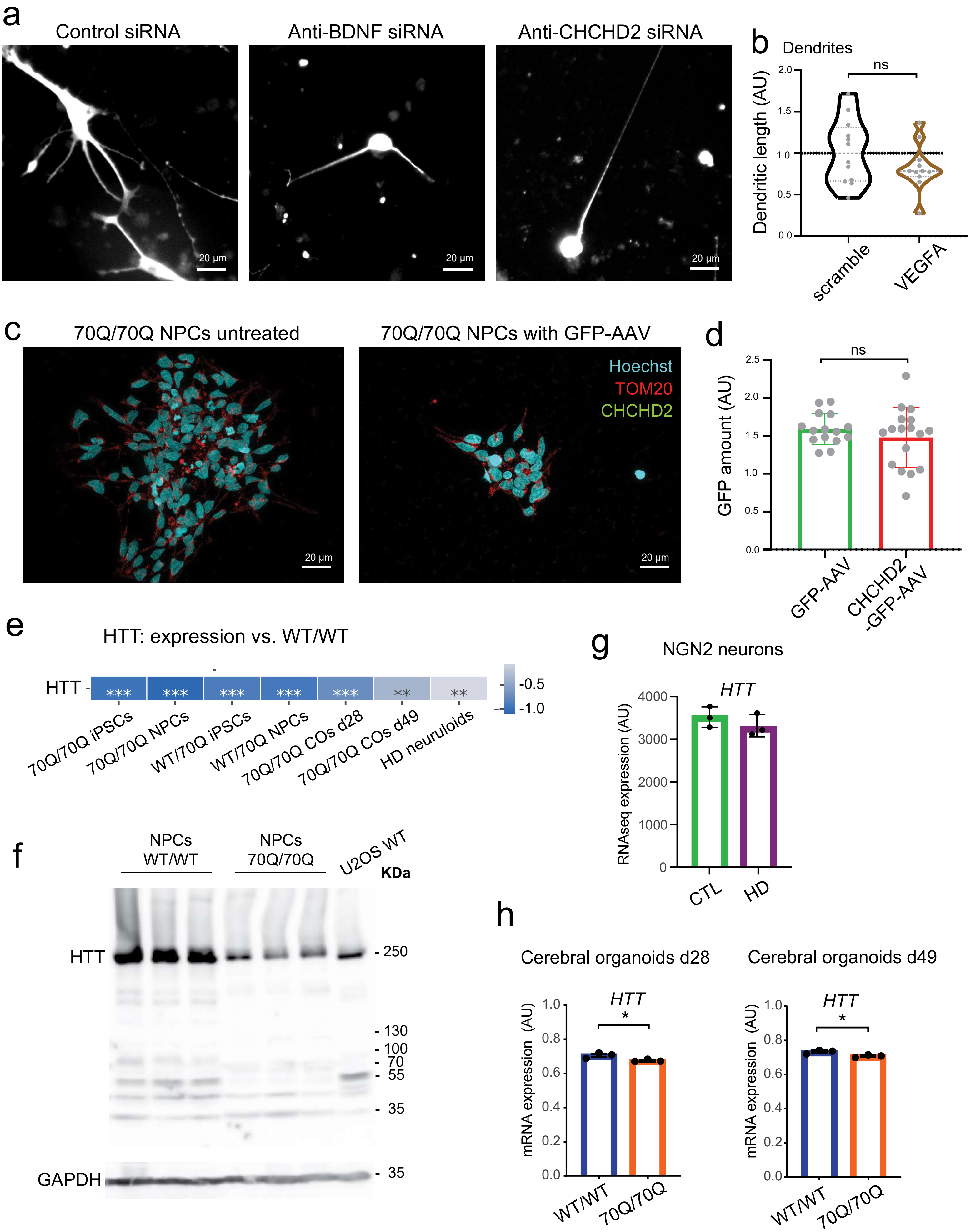
a
Control siRNA
Anti-BDNF siRNA
Anti-CHCHD2 siRNA
20 µm
b
Dendrites
Dendritic length (AU)
ns
2.0
1.5
1.0
0.5
0.0
scramble
VEGFA
c
70Q/70Q NPCs untreated
70Q/70Q NPCs with GFP-AAV
Hoechst
TOM20
CHCHD2
d
GFP amount (AU)
ns
2.5
2.0
1.5
1.0
0.5
0.0
GFP-AAV
CHCHD2 -GFP-AAV
e
HTT: expression vs. WT/WT
HTT
***
**
-0.5
-1.0
70Q/70Q iPSCs
70Q/70Q NPCs
WT/70Q iPSCs
WT/70Q NPCs
70Q/70Q COs d28
70Q/70Q COs d49
HD neuruloids
f
NPCs WT/WT
NPCs 70Q/70Q
U2OS WT
KDa
HTT
250
130
100
70
55
35
GAPDH
35
g
NGN2 neurons
HTT
RNAseq expression (AU)
4000
3000
2000
1000
0
CTL
HD
h
Cerebral organoids d28
Cerebral organoids d49
HTT
*
mRNA expression (AU)
1.0
0.8
0.6
0.4
0.2
0.0
WT/WT
70Q/70Q

Supplementary Fig. 9. Manipulation of CHCHD2 expression in neural cells (related to Fig. 7). a Representative images of NGN2 neurons directly generated from the healthy inducible iPSC line BIHi005-A visualized at day 17, four days after transfection with different siRNA constructs. NGN2 neurons transfected with scramble control siRNA exhibit multiple neurite branching, while these were diminished in NGN2 neurons transfected with siRNA against BDNF or CHCHD2. Data were repeated in three independent experiments. **b** Quantification of neuronal arborization assessed by high-content imaging based on antibodies labeling dendrites (MAP2) at day 17, two days after transfection with either control scramble siRNA or siRNA against VEGFA. Dot indicates different replicates out of two independent experiments. ns: not significant; unpaired two-tailed Welch t test. **c** Representative images of 70Q/70Q NPCs at basal condition or after transduction with GFP-AAV showing similar level of TOM20 and CHCHD2 expression. Data were repeated in two independent experiments. **d** Quantification of GFP signal amount in 70Q/70Q NPCs transduced with either GFP-AAV or CHCHD2-GFP-AAV. Dots represent individual images collected from two independent experiments. ns: not significant; unpaired two-tailed Welch t test. **e** Heatmap of log-fold change (LFC) comparisons of *HTT* gene based on bulk RNA sequencing performed in iPSCs, NPCs, and COs from 70Q/70Q and WT/70Q compared to WT/WT. HD neuruloids were compared to WT neuruloids. **f** Representative immunoblot of wild-type HTT in NPCs from WT/WT and 70Q/70Q. The results were confirmed in two independent experiments. **g** *HTT* expression based on RNA sequencing in NGN2 neurons derived from controls (C1, C2, C3) and individuals with HD (HD1, HD2, HD3) (AU=arbitrary units). **h** qPCR-based *HTT* expression in unguided cerebral organoids at day 28 and 49. Mean +/- s.e.m.; n=3 independent biological replicates (dots) per line (AU=arbitrary units); *p<0.05 **p<0.01; unpaired two-tailed t test. Four organoids were pooled for each individual RNA isolation.

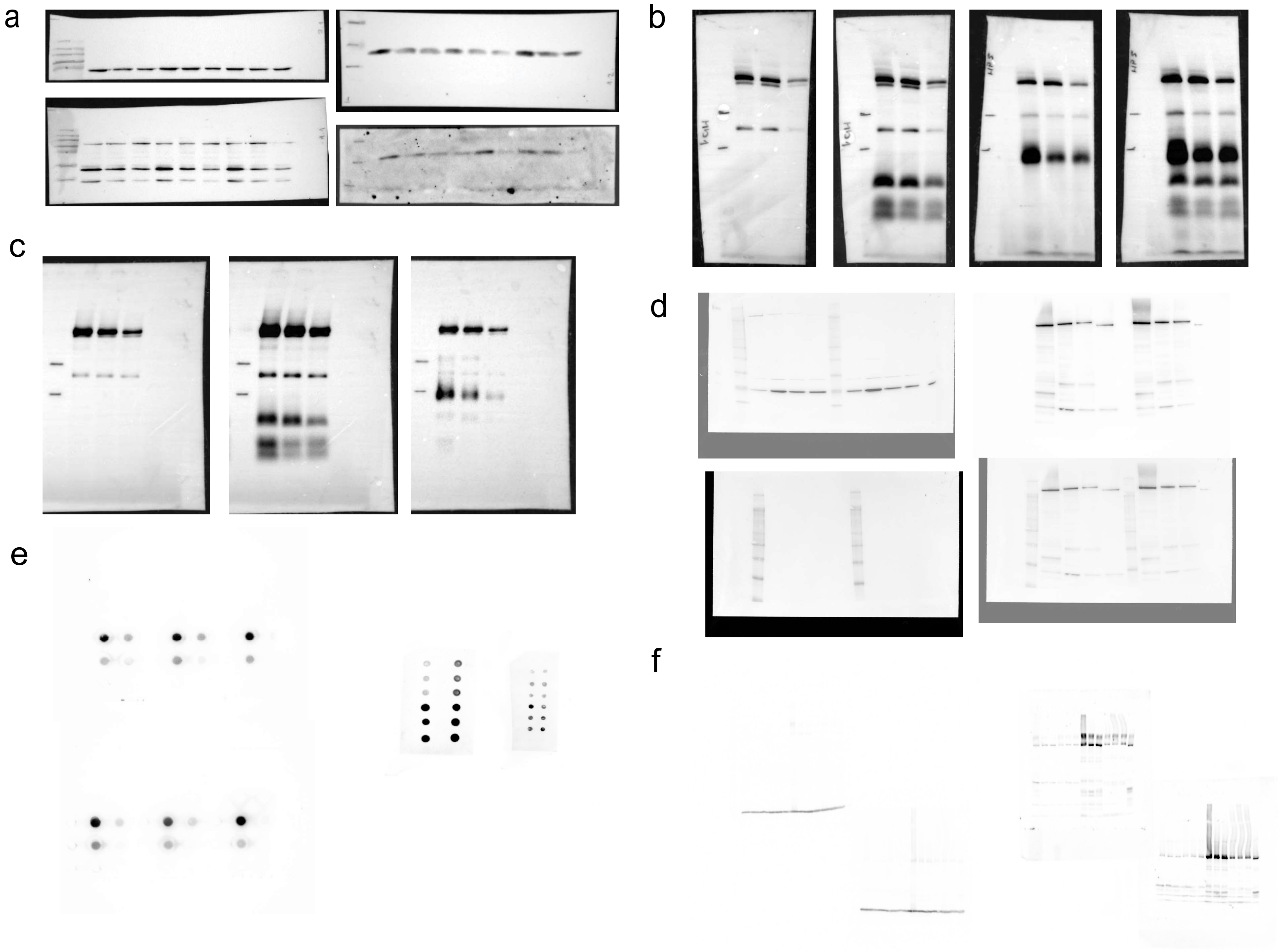
a
b
c
d
e
f

Supplementary Fig. 10. Original blots (related to Fig. 5, Supplementary Fig. 1, Supplementary Fig. 2, Supplementary Fig. 6, Supplementary Fig. 9). a Unprocessed blot scans related to Fig. 5g. **b** Unprocessed blot scans related to Fig. 5i. **c** Unprocessed blot scans related to Supplementary Fig. 6h. **d** Unprocessed blot scans related to Supplementary Fig. 1b. **e** Unprocessed blot scans related to Supplementary Fig. 2c. **f** Unprocessed blot scans related to Supplementary Fig. 9f.
