# Supplementary material for: Mutant huntingtin impairs neurodevelopment in human brain organoids through CHCHD2-mediated neurometabolic failure
Source: Nat Commun. 2024 Aug 22;15:7027. doi: 10.1038/s41467-024-51216-w (PMC11341898; doi:10.1038/s41467-024-51216-w)
Supplement: Supplementary file 3 — Description of Additional Supplementary Files [file 41467_2024_51216_MOESM3_ESM.pdf]

**Description of Additional Supplementary Files**

**Supplementary Data 1.** Genes expressed by the four populations identified by single-cell RNA sequencing analysis of midbrain organoids from WT/WT and 70Q/70Q (related to Fig. 2 and Supplementary Fig. 3).

**Supplementary Data 2.** Differentially expressed genes (DEG) in iPSCs, NPCs, cerebral organoids (COs) at d28 and CO at d49 derived from WT/WT and 70Q/70Q (related to Fig. 3 and Supplementary Fig. 4).

**Supplementary Data 3.** Common genes expressed in iPSCs, NPCs, cerebral organoids (COs) at d28 and CO at d49 derived from WT/WT and 70Q/70Q (related to Fig. 3 and Supp Fig. 4).

**Supplementary Data 4.** Proteomics: differentially expressed proteins in iPSCs 70Q/70Q compared to iPSCs WT/WT (two-tailed t-test with Benjamini-Hochberg correction for multiple comparisons; FDR<0.05) (related to Figure 3).

**Supplementary Data 5.** Proteomics: differentially expressed proteins in NPCs 70Q/70Q compared to NPCs WT/WT (two-tailed t-test with Benjamini-Hochberg correction for multiple comparisons; FDR<0.05) (related to Figure 3).

**Supplementary Data 6.** Functional metabolic analysis from proteomics of iPSCs 70Q/70Q compared to iPSCs WT/WT and NPCs 70Q/70Q compared to NPCs WT/WT (related to Fig. 5).

**Supplementary Data 7.** Total RNA-sequencing: differentially expressed genes and pathways in NGN2 neurons from healthy controls (C1, C2, C3) and individuals with HD (HD1, HD2, HD3) (two-

tailed Wald test with Benjamini-Hochberg correction for multiple comparisons; FDR<0.05) (related to Fig. 6 and Supplementary Fig. 7).

**Supplementary Data 8.** Proteomics: differentially expressed proteins in NGN2 neurons from healthy controls (C1, C2, C3) and individuals with HD (HD1, HD2, HD3) (two-tailed Wald test with Benjamini-Hochberg correction for multiple comparisons; FDR<0.05) (related to Fig. 6 and Supplementary Fig. 7).

**Supplementary Data 9.** Proteomics: gene set enrichment analysis (GSEA) in NGN2 neurons from healthy controls (C1, C2, C3) and individuals with HD (HD1, HD2, HD3) (two-tailed Wald test with Benjamini-Hochberg correction for multiple comparisons; FDR<0.05) (related to Fig. 6 and Supplementary Fig. 7).

**Supplementary Data 10.** Metabolomics: differentially expressed metabolites in NGN2 neurons from healthy controls (C1, C2, C3) and individuals with HD (HD1, HD2, HD3) (two-tailed Wald test with Benjamini-Hochberg correction for multiple comparisons; FDR<0.05) (related to Fig. 6 and Supplementary Fig. 7).

**Supplementary Data 11.** List of sequences and primers for PCR, QRT-PCR, siRNA knockdown, and CRISRP/eCas9 genome editing, and list of all antibodies.
