# Supplementary material for: Mutant huntingtin impairs neurodevelopment in human brain organoids through CHCHD2-mediated neurometabolic failure
Source: Nat Commun. 2024 Aug 22;15:7027. doi: 10.1038/s41467-024-51216-w (PMC11341898; doi:10.1038/s41467-024-51216-w)
Supplement: Supplementary file 15 — Reporting Summary [file 41467_2024_51216_MOESM15_ESM.pdf]

nature portfolio

Corresponding author(s): Alessandro Prigione

Last updated by author(s): Jul 23, 2024

Reporting Summary

Nature Portfolio wishes to improve the reproducibility of the work that we publish. This form provides structure for consistency and transparency in reporting. For further information on Nature Portfolio policies, see our Editorial Policies and the Editorial Policy Checklist.

Statistics

For all statistical analyses, confirm that the following items are present in the figure legend, table legend, main text, or Methods section.

n/a	Confirmed	
☐	☒	The exact sample size (*n*) for each experimental group/condition, given as a discrete number and unit of measurement
☐	☒	A statement on whether measurements were taken from distinct samples or whether the same sample was measured repeatedly
☐	☒	The statistical test(s) used AND whether they are one- or two-sided<br>*Only common tests should be described solely by name; describe more complex techniques in the Methods section.*
☒	☐	A description of all covariates tested
☒	☐	A description of any assumptions or corrections, such as tests of normality and adjustment for multiple comparisons
☐	☒	A full description of the statistical parameters including central tendency (e.g. means) or other basic estimates (e.g. regression coefficient) AND variation (e.g. standard deviation) or associated estimates of uncertainty (e.g. confidence intervals)
☐	☒	For null hypothesis testing, the test statistic (e.g. *F*, *t*, *r*) with confidence intervals, effect sizes, degrees of freedom and *P* value noted<br>*Give P values as exact values whenever suitable.*
☒	☐	For Bayesian analysis, information on the choice of priors and Markov chain Monte Carlo settings
☒	☐	For hierarchical and complex designs, identification of the appropriate level for tests and full reporting of outcomes
☒	☐	Estimates of effect sizes (e.g. Cohen's *d*, Pearson's *r*), indicating how they were calculated

Our web collection on statistics for biologists contains articles on many of the points above.

Software and code

Policy information about availability of computer code

Data collection	Bulk RNAseq, single-cell RNAseg, long-read RNAseq, Mass-spectrometry (MS)-based proteomics and metabolomics analysis, immunoblots, images acquisition, HCA, bioenenergetics. No software were used for other data collection.
Data analysis	Softwares for data analysis: ImageJ (v. 1.53a), GraphPad-Prism (v. 10.0), KaryoStudio (v. 1.4), CellProfiler (v. 4.2.1), Inkscape(v. 1.2.1), Zeiss blue software (v. 3.1).<br>Softwares for omics analysis: Cell Ranger (v. 7.1.0), R (v. 4.2.2), edgeR (v 3.40.2), “Sublime Text” for Windows (Version Build 4113), ggplot2 (v. 3.5.0), dplyr (v. 1.1.4), Reactome (v. 7.2), KEGG (v. 7.2), Bioconducter clusterProfiler (v.3.0.4), Gephi (v. 0.10.1), MetaboAnalyst platform (v. 5.0), OmicsNet platform (v. 2.0), Cytoscape (v. 3.9.1), MultiQuantTM (v.2.1.1), MaxQuant software (v1.6.10.43).<br>Code for sRNAseq data analysis: https://github.com/rajewsky-lab/Huntington_midbrain_organoids.<br>Code for CHCHD2 and TOM20 quantification analyses: https://github.com/Scaramir/HD_colocalization.<br>Pipeline for the high-content analysis (HCA) of neurite outgrowth with the open-source software CellProfiler: https://zenodo.org/records/6642365.

For manuscripts utilizing custom algorithms or software that are central to the research but not yet described in published literature, software must be made available to editors and reviewers. We strongly encourage code deposition in a community repository (e.g. GitHub). See the Nature Portfolio guidelines for submitting code & software for further information.

Data

Policy information about availability of data

All manuscripts must include a data availability statement. This statement should provide the following information, where applicable:

- Accession codes, unique identifiers, or web links for publicly available datasets
- A description of any restrictions on data availability
- For clinical datasets or third party data, please ensure that the statement adheres to our policy

There are restrictions to the availability of the patient-derived iPSC lines due to the nature of our ethical approval that does not support sharing to third parties and does not allow to perform genomic studies to respect the European privacy protection law. The datasets generated during this study are available, in cases data protection laws did not prevent the original datasets from being published.
The RNA-sequencing data generated in this study have been deposited in the Gene Expression Omnibus (GEO) database: under accession code GSE233916:
The mass spectrometry proteomics data generated in this study have been deposited in the ProteomeXchange Consortium via the PRIDE partner repository under the accession code PXD041846: http://www.ebi.ac.uk/pride/archive/projects/PXD041846.
The mass spectrometry metabolomics data generated in this study have been deposited in Peptide Atlas repository under the accession code PASS04827: http://www.peptideatlas.org/PASS/PASS04827.

Research involving human participants, their data, or biological material

Policy information about studies with human participants or human data. See also policy information about sex, gender (identity/presentation), and sexual orientation and race, ethnicity and racism.

Reporting on sex and gender	Engineered iPSCs (70Q/70Q, WT/70Q, 0Q/0Q) were derived in the male background, and the three patient HD lines (HD1-3) and three control lines (C1-3) are all male. Therefore, we will avoid confounding effects due to potential sex-related differences.
Reporting on race, ethnicity, or other socially relevant groupings	We do not know the identity of the 3 patients from we which derived iPSCs, as the fibroblasts were collected in anonymous manner.
Population characteristics	We do not know the identity of the 3 patients from we which derived iPSCs as the fibroblasts were collected in anonymous manner.
Recruitment	We did not recruit patients.
Ethics oversight	Ethical approval for using iPSCs from HD individuals and control subjects was obtained from the Ethic Committee of the University Clinic Düsseldorf (study number 2019-681 approved on October 11, 2019).

Note that full information on the approval of the study protocol must also be provided in the manuscript.

Field-specific reporting

Please select the one below that is the best fit for your research. If you are not sure, read the appropriate sections before making your selection.

☒ Life sciences ☐ Behavioural & social sciences ☐ Ecological, evolutionary & environmental sciences

For a reference copy of the document with all sections, see nature.com/documents/nr-reporting-summary-flat.pdf

Life sciences study design

All studies must disclose on these points even when the disclosure is negative.

Sample size	We chose the sample size based on previous experience and in accordance to the standards in the field. of iPSC-based disease modeling. We used independent control lines and isogenic control lines to increase the robustness of the results. For patient-derived lines, we included three patients and three control lines that were matched for age and gender. We performed all in vitro experiments using at least three biological replicates over different independent experiments.
Data exclusions	No data was excluded from the analyses. We performed outlier test analysis to identity potentail outliers using GraphPad: https://www.graphpad.com/quickcalcs/Grubbs1.cfm.
Replication	We repeated all experiments using at least three biological replicates and several technical replicates over distinct independent experiments. We specified the number of replicates and independent experiments in the respective figure legends.
Randomization	We plated the cells in a random distribution onto cell culture and multi-well plate positions, and randomly assigned them to experimental groups. We perfomed cell counting on random miscroscope view fields. We then grouped the samples based on their genotypes or the specific treatments, and we compared them to the respective control groups.
Blinding	The investigators who performed the RNA sequencing, proteomics, metabolomis, omics integration analysis, and immuboblot staining were

Blinding | blinded to the genotypes of groups and samples. In other cases it was not possible for the investigators to be blinded as the patient samples showed easily distinguishable behaviors.

Reporting for specific materials, systems and methods

We require information from authors about some types of materials, experimental systems and methods used in many studies. Here, indicate whether each material, system or method listed is relevant to your study. If you are not sure if a list item applies to your research, read the appropriate section before selecting a response.

Materials & experimental systems

n/a	Involved in the study
☐	☒ Antibodies
☐	☒ Eukaryotic cell lines
☒	☐ Palaeontology and archaeology
☒	☐ Animals and other organisms
☒	☐ Clinical data
☒	☐ Dual use research of concern
☒	☐ Plants

Methods

n/a	Involved in the study
☒	☐ ChIP-seq
☒	☐ Flow cytometry
☒	☐ MRI-based neuroimaging

Antibodies

Antibodies used

CHCHD2 rabbit 1:800 Atlas Antibodies HPA027407
TOM20 mouse 1:500 MilliporeSigma MABT166
TUJ1 mouse 1:2000 Sigma-Aldrich, T8578-100UL
SOX2 Goat 1:100 Santa Cruz Biotechnology sc-17320
FOXA2 rabbit 1:300 Sigma-Aldrich HPA066846
DARPP-32 goat 1:50 Santa Cruz Biotechnology sc-31518
TOM20 rabbit 1:200 Santa Cruz Biotechnology sc-11415
GFAP guiney pig 1:500 Synaptic Systems 173 004
SMI312 mouse 1:500 BioLegend 837904
MAP2 guiney pig 1:1000 Synaptic Systems 188 004
EM48 mouse 1:1000 MilliporeSigma MAB5374
Tau rabbit 1:500 Sigma-Aldrich SAB4501831
Nestin mouse 1:200 MilliporeSigma MAB5326
PAX6 rabbit 1:200 BioLegend 901301
CTIP2 rabbit 1:300 Abcam ab240636
FOXG1 rabbit 1:500 Abcam ab18259
CHCHD2 rabbit 1:800 Atlas Antibodies HPA027407
ZO1 mouse 1:200 Thermo Fisher Scientific 33-9100
CTIP2 rabbit 1:300 Abcam ab240636
SOX2 goat 1:100 Santa Cruz Biotechnology sc-17320
ZO1 mouse 1:200 Thermo Fisher Scientific 33-9100
TH Rabbit 1:500 Millipore AB152
MAP2 guiney pig 1:1000 Synaptic Systems 188 004
SOX2 Goat 1:100 Santa Cruz Biotechnology sc-17320
NGN2 neurons BIHi005-A-24
MAP2 guinea pig 1:1000 Synaptic Systems 188 004
SMI312 mouse 1:500 BioLegend 188 004
NDUFA9 mouse SDS: 1:1000 Abcam Cat#ab14713; RRID:AB_301431
BNE: 1:1000
NDUFV1 rabbit SDS: 1:1000 Proteintech Cat#11238-1-AP; RRID: AB_2149040
SDHA mouse SDS: 1:10 000 Abcam Cat#ab14715; RRID:AB_301433
BNE: 1:10000
UQCRC2 (CORE2) mouse SDS: 1:1000 Abcam Cat#ab14745; RRID:AB_2213640
BNE: 1:1000
CYC1 rabbit SDS: 1:1000 Merck / Sigma Aldrich Cat#HPA001247; RRID:AB_1078602
COX5A mouse SDS: 1:1000 Abcam Cat# ab110262; RRID: AB_10861723
COX5B mouse SDS: 1:1000 Santa Cruz Biotechnology Cat#sc-374417; RRID: AB_10988066
BNE: 1:1000
NDUFA4 rabbit SDS: 1:1000 Abcam Cat#ab129752; RRID:AB_11155881
HTT Rabbit monoclonal 1:1000 Abcam ab109115
GAPDH Mouset monoclonal 1:500 Santa Cruz sc-47724
ACTB Mouse monoclonal 1:5000 Becton Dickinson 612656

Validation

All antibodies used in this study are commercial and well-established in the field. Validation data are provided for each antibody by the manufacturer. In addition, we validated the antibodies for staining and immunoblotting using multiple control-derived samples prior to performing comparative analyses.

Eukaryotic cell lines

Policy information about cell lines and Sex and Gender in Research

Cell line source(s)	WT/WT iPSCs for genome editing were derived using Sendai viruses from a healthy middle-aged male individual (BIHi050-A/SCVI113). iPSCs from three male individuals with HD were described before: HD1 with WT/180Q (BIHi035-A), HD2 with WT/58Q (BIHi288-A), and HD3 with WT/44Q (BIHi033-A). Control iPSCs used for comparison with HD iPSCs were derived before: C1 (TFBJ, HHUUKDi009-A), C2 (XM001, BIHi043-A), and C3 (BIHi005-A). For engineering an inducible NGN2 iPSC line, we used the control healthy iPSC line C3 (BIHi005-A). HEK 293 cells were used for NGN2 lentiviral generation.
Authentication	We authenticated all iPSC lines (from control subjects, patients, and genetically modified) using STR analysis and SNP karyotyping. No authentication was performed for HEK 293 cells, as they were only used to generate lentiviruses for induction of NGN2 neurons.
Mycoplasma contamination	All cells tested negative for mycoplasma contamination based on continuous routine analyses.
Commonly misidentified lines (See ICLAC register)	No commonly misidentified lines were used.

Plants

Seed stocks	*Report on the source of all seed stocks or other plant material used. If applicable, state the seed stock centre and catalogue number. If plant specimens were collected from the field, describe the collection location, date and sampling procedures.*
Novel plant genotypes	*Describe the methods by which all novel plant genotypes were produced. This includes those generated by transgenic approaches, gene editing, chemical/radiation-based mutagenesis and hybridization. For transgenic lines, describe the transformation method, the number of independent lines analyzed and the generation upon which experiments were performed. For gene-edited lines, describe the editor used, the endogenous sequence targeted for editing, the targeting guide RNA sequence (if applicable) and how the editor was applied.*
Authentication	*Describe any authentication procedures for each seed stock used or novel genotype generated. Describe any experiments used to assess the effect of a mutation and, where applicable, how potential secondary effects (e.g. second site T-DNA insertions, mosiacism, off-target gene editing) were examined.*
